# Demethylzeylasteral inhibits oxidative phosphorylation complex biogenesis by targeting LRPPRC in lung cancer

**DOI:** 10.7150/jca.92797

**Published:** 2025-01-01

**Authors:** Lina Wang, Wei Zhou, Wenxi Wang, Yuxin Liang, Qiqi Xue, Zhen Zhang, Jinghe Yuan, Xiaohong Fang

**Affiliations:** 1 Key Laboratory of Molecular Nanostructure and Nanotechnology, CAS Research/Education Center for Excellence in Molecular Sciences, Institute of Chemistry, Chinese Academy of Science, Beijing 100190, PR China.; 2 University of the Chinese Academy of Sciences (UCAS), Beijing 100049, PR China.; 3 Hangzhou Institute of Medicine (HIM), University of Chinese Academy of Sciences (Zhejiang Cancer Hospital), Chinese Academy of Sciences, Hangzhou, Zhejiang 310022, PR China.

**Keywords:** Traditional Chinese Medicine (TCM), Demethylzeylasteral (T-96), LRPPRC, OXPHOS, lung cancer

## Abstract

Targeted inhibition of mitochondrial oxidative phosphorylation (OXPHOS) complex generation is an emerging and promising cancer treatment strategy, but limited targets and specific inhibitors have been reported. Leucine-rich pentatricopeptide repeat-containing protein (LRPPRC) is an atypical RNA-binding protein that regulates the stability of all 13 mitochondrial DNA-encoded mRNA (mt-mRNA) and thus participates in the synthesis of the OXPHOS complex. LRPPRC is also a prospective therapeutic target for lung adenocarcinoma, serving as a promising target for OXPHOS inhibition. In this study, we identified Demethylzeylasteral (T-96), a small molecule extracted from the Chinese herb *Tripterygium wilfordii* Hook. f., as a novel inhibitor of LRPPRC. T-96 directly bound to the RNA-binding domain of LRPPRC, inhibiting its interaction with mt-mRNA. This led to instability in both mt-mRNA and LRPPRC protein. Treatment with T-96 significantly reduced the mRNA and protein levels of the OXPHOS complex. As a consequence of LRPPRC inhibition, T-96 treatment induced a defect in the synthesis of the OXPHOS complex, inhibiting mitochondrial aerobic respiration and ATP synthesis. Moreover, T-96 exhibited potent antitumor activity for lung adenocarcinoma *in vitro* and *in vivo*, and the antitumor effect of T-96 was dependent on LRPPRC expression. In conclusion, this study not only identified the first traditional Chinese medicine monomer inhibitor against OXPHOS complex biosynthesis as well as a novel target of Demethylzeylasteral, but also shed light on the unique antitumor mechanism of bioactive compounds derived from traditional Chinese medicine.

## Introduction

All biological processes of cancer cells, including proliferation, metastasis, and relapse, are fueled by metabolism. Since the discovery of the Warburg effect in the 1920s, glycolysis has been regarded as the preferable metabolic pathway for tumors^1^. However, the growing body of evidence indicates a significant role of mitochondrial oxidative phosphorylation (OXPHOS) in tumor progression^2^-^4^. It has been documented that cancer cells possess robust mitochondrial function in which OXPHOS generates ATP and various nutrient molecules to support the growth of tumor cells^2^. In addition, cells responsible for tumor metastasis or drug resistance are more likely to utilize OXPHOS^5^. Furthermore, numerous studies have demonstrated that cancer stem cells, which are accountable for tumor metastasis and recurrence, favor OXPHOS over glycolysis^6^-^8^. The indispensable functions of OXPHOS in tumor proliferation, metastasis, drug tolerance, and tumor recurrence indicate that targeting OXPHOS is a promising strategy for treating tumors^9^-^11^.

Until now, efforts in OXPHOS-targeted therapy have focused primarily on directly inhibiting the activity of OXPHOS complexes, such as oligomycin^12^ and rotenone^13^, ^14^. The deleterious side effects on normal cells are inevitable due to the reliance of non-neoplastic cells on OXPHOS^15^. Another emerging concept aims to inhibit mitochondrial OXPHOS complex generation without inhibiting the existing OXPHOS complex's activity. Since maintaining mitochondrial mass homeostasis requires rapid synthesis of the OXPHOS complex in rapidly replicating cancer cells, such inhibitors can selectively inhibit the mitochondrial function of tumor cells. Consequently, this induces an energy crisis within the cancer cells while exerting minimal impact on slowly proliferating normal cells. Therefore, downregulation of mitochondrial gene expression has been reported to realize this concept^16^, ^17^. Mitochondrial DNA (mtDNA) encodes thirteen proteins, all of which are essential components of the OXPHOS complexes. It is known that POLRMT transcribes mtDNA into mitochondrial RNA (mtRNA), which further interacts with LRPPRC to promote mt-mRNA maturation and stability. Mitochondrial ribosome translates mt-mRNAs into proteins to form the OXPHOS complexes^18^. Efforts have been made to inhibit POLRMT (IMT)^16^, ^19^, ^20^ and mitochondrial ribosome (tigecycline)^21^. While there is ongoing debate regarding the functions of POLRMT in tumor progression, and tigecycline is strictly restricted for preventing the spread of superresistant bacteria^17^, it is reasonable to explore a new approach to target LRPPRC for OXPHOS inhibition.

Traditional Chinese Medicine (TCM) has been used extensively in China for thousands of years to treat a variety of diseases. It has become an invaluable resource for the discovery of novel active drug monomers as a result of its extensive clinical experience. Up to now, no traditional Chinese medicine monomer with the ability to inhibit OXPHOS complex formation has been reported. In our previous research, we identified LRPPRC as a druggable target in various cancers^22^. Our aptamer-based high-throughput drug screening system suggested that the Demethylzeylasteral (T-96), a triterpene compound extracted from the commonly used Chinese herbal medicine *Tripterygium wilfordii* Hook. f. in clinics, is a potential inhibitor of LRPPRC^22^. It has been reported that Demethylzeylasteral possesses a variety of pharmacological effects, including anticancer, anti-inflammatory, immune suppression, anti-fertility, antivirus, and antimicrobial activities. Meanwhile, research has also demonstrated that Demethylzeylasteral can target multiple critical signaling pathways within cells to exert its effects. These pathways include the TGF-β signaling pathway^23^, FBXW7/c-Myc axis^24^, miR-30e-5p/MYBL2 axis^25^, extrinsic apoptosis pathway^26^ and others. However, the direct binding target of Demethylzeylasteral remains unclear, which restricts the precision medication for T-96.

In this study, we demonstrated that the RNA binding protein LRPPRC is a direct binding target of T-96, which is important for its antitumor function. Mechanistically, T-96 bound to the RNA-binding domain of LRPPRC directly and inhibited the nucleic acid binding ability of LRPPRC. Administration of T-96 suppressed the interaction between LRPPRC and mt-mRNA, inducing the instability of both mt-mRNA and LRPPRC. This led to a decreased expression of OXPHOS complexes, resulting in further mitochondrial synthetic defect and an energy crisis. Our study achieved the suppression of OXPHOS complex formation using a traditional Chinese medicine monomer. Additionally, our findings revealed a novel molecular mechanism by which the traditional Chinese medicine monomer suppresses tumors, providing a theoretical basis for future clinical treatment.

## Material and Methods

### Reagent

Demethylzeylasteral (T-96, CAS: 107316-88-1) was purchased from Selleck (S3608) and dissolved in DMSO as a 50 mM stock solution.

### Cell culture

All cells were purchased from the Chinese National Infrastructure of Cell Line Resource (Beijing, China). A549 (RRID: CVCL_0023) were cultured in a DMEM (Gibco, USA) medium containing 10% fetal bovine serum (FBS) and 1% penicillin/streptomycin (Gibco, USA). H1299 (RRID: CVCL_0060) were cultured in RPMI 1640 medium containing 10% FBS (Gibco, USA) and 1% penicillin-streptomycin (Gibco, USA). All experiments were performed with mycoplasma-free cells.

### Cell viability assay

A549 cells were plated into 96-well plates (2,000 cells per well) and cultured with different concentrations of T-96 (32 μM, 16 μM, 8 μM, 4 μM, 2 μM, 1 μM, 0.5 μM, 0 μM). After 48 h and 72 h respectively, cell viability was assessed using the Cell Counting Kit-8 (CCK8, DOJINDO) according to the manufacturer's instructions. Briefly, each well was supplemented with 10 µL of CCK8 solution and incubated for an additional hour. Then the absorbance at 450 nm was determined using a spectrophotometer. Wells without any cells served as controls.

### Apoptosis assays

Cell apoptosis was validated with Annexin V-FITC/PI Kit (FXP018-050, 4A Biotech) according to the manufacturer's protocol. Briefly, A549 cells (10^5^ cells/well) were seeded in six-well plates and treated with or without T-96 for 24 h, 48 h, 72 h, and 96 h. Cells were washed twice with cold PBS and then resuspended in 100 μL 1×Binding buffer. After adding 5 μL of Annexin V-FITC, the cells were gently vortexed and incubated for 5 min at room temperature in the dark. Subsequently, 10 μL a 20 μg/mL PI solution and 400 μL DPBS were added to each sample. The samples were then immediately tested using flow cytometry with a BD Accuri C6, and the results were analyzed with FlowJo V10 software.

### Colony formation assay

A549 cells were plated in 12-well plates (10^4^ cells/well) and cultured for 7 days under different T-96 treatment conditions (0, 2.5 μM, 5 μM, 10 μM). The colonies that had formed were rinsed twice with PBS, fixed with 4% Paraformaldehyde for 20 min at room temperature, and then stained with 1% crystal violet for 30 min. All statistical measurements were performed in triplicate.

### Scratch wound healing assay

A549 cells were cultured in plates until they reached 80% confluence. Scratch wounds were created using a 200-µL pipette tip, and cell debris was removed using PBS. Subsequently, the cells were treated with T-96 (0, 2.5 μM, 5 μM) in DMEM medium containing 10% FBS for 24 h and 48 h. Cell migration was assessed at 0 h, 24 h, and 48 h. The migrated areas were analyzed using ImageJ software.

### Transwell invasion assay

Transwell chambers (Corning) were placed in 24-well plates. Matrigel (356234, Corning, USA) was diluted to a concentration of 200 µg/mL, and 150 µL of the diluted Matrigel gel was added to each upper chamber. After the gel solidified, A549 cells in the logarithmic growth phase were collected and diluted to 5×10^5^ cells/mL in serum-free DMEM medium. The upper chambers were given 150 µL of cell suspension, and the lower chambers were given 800 µL of DMEM medium containing 20% FBS. After incubating for 24 h at 37 °C, the cells were rinsed twice with PBS. The residual Matrigel in each chamber was wiped off with a cotton swab. After fixing with 4% paraformaldehyde for 20 min and staining with crystal violet for 15 min at room temperature, they were imaged under a microscope. The experiment was repeated three times.

### GST fusion protein and biotin-labeled protein preparation

The DNA sequence of the LRPPRC fragment (LRPPRCΔ5, 1121aa-1394aa) was inserted into the pGEX-4T1 plasmids and the expression of GST fusion protein in *E.coli* BL21 (DE3) was induced at 27 °C by 1 mM isopropyl-β-d-thiogalactoside (IPTG)^22^. The bacteria were centrifuged at 8000×g for 15 min at 4 °C to remove supernatant and subsequently lysed by lysozyme for 30 minutes on ice. The GST fusion proteins were then purified according to the manufacturer's guidelines using GST 4FF (Pre-Packed Gravity Column, C600913, Sangon Biotech) and concentrated utilizing an ultrafiltration device with a molecular weight cutoff of 30 kDa. LRPPRC∆5 was biotinylated by incubating it with NHS-PEG_12_-Biotin for 30 minutes at room temperature, followed by the removal of excess biotinylation reagent with PD MiniTrap^TM^ G-25 Desalting Column (28918007, GE Healthcare). All the purified proteins were dissolved in DPBS buffer, flash-frozen in liquid nitrogen, and stored at -80 °C until use.

### Fluorescence titration

Fluorescence measurements were conducted using the FLS980 fluorescence spectrometer (Edinburgh Instruments, Ltd.) using a 5-mm quartz cuvette. A stock solution of Demethylzeylasteral (T-96) at a concentration of 50 mM was prepared in DMSO and then diluted to 64 μM, 32 μM, 16 μM, 8 μM, 4 μM, 2 μM, and 1 μM in DPBS. Each dilution was mixed with an equal volume of LRPPRC at a concentration of 240 μg/mL LRPPRC. Intrinsic fluorescence emission spectra of 120 μL LRPPRC in the presence of different concentrations of T-96 (32 μM, 16 μM, 8 μM, 4 μM, 2 μM, 1 μM, 0.5 μM) were performed in the DPBS at room temperature. The excitation wavelength was fixed at 280 nm for this purpose, and emission spectra were collected in the range of 300-400 nm. The excitation and emission slit widths (1 nm each) were constantly maintained for all experiments. A typical emission peak was observed at 315 nm. The final spectrum was obtained after subtracting their corresponding blank spectra. Three independent experiments were performed for each molecule, and their average values were used for data analyses. A decrease in the fluorescence intensity resulting from increasing concentrations of ligands was employed as a parameter to calculate the binding constant 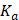 and the number of binding sites 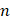 using the modified Stern-Volmer equations with slight modification^27^.


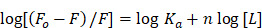
, (1)

where 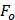 is the fluorescence intensity of protein, 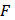
is the fluorescence intensity of ligand, 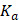 is the binding constant, 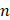 is the number of binding sites, and 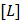 represents ligand concentration. For the ligand-protein complex, the values for 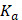
and 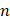 can be derived from the intercept and slope, respectively.

### Biolayer interferometry (BLI)

BLI was performed on an Octet RED96 system (ForteBio, Molecular Devices) with shaking at 1000 rpm. The BLI assay buffer consisted of 0.1% BSA in PBSP (0.02% P20), which was filtered through a 0.22 μm filter. Streptavidin Dip and Read^TM^ Biosensors (18-5019, ForteBio, Molecular Devices) were loaded into the columns of a biosensor holding plate and pre-hydrated in BLI assay buffer for 10 minutes before use. Each well of a 96-well slant-bottom microplate was loaded with 200 μL of sample. The assay plate was prepared as follows: column1 (BLI assay buffer), column2 (5 mg/mL biotin-labeled LRPPRC
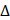
5 in BLI assay buffer), column3 (10 μg/mL biocytin), column4 (BLI assay buffer), column5 (BLI assay buffer and 50 μM, 100 μM, 150 μM, and 200 μM T-96 in BLI assay buffer). The double reference subtraction method is set up as follows: Baseline (120 s) in column1 (Equilibration), Loading (300 s) in column2 (Ligand sensors) or column1 (Reference sensors), Quenching (60 s) in column3, Baseline (120 s) in column4 (Wash), Association (60 s) in column5. Dissociation (180 s) in column 4. Loading of biotin-labeled LRPPRC
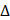
5 300 s onto SA sensor tips resulted in a wavelength shift signal of ~3.7 nm. The data underwent double reference subtraction, and the plots were fitted with a 2:1 model using the Pall FortéBio analysis software (version 11.0.0.4).

### Cellular Thermal Shift Assay (CETSA)

The CETSA experiment was performed using A549 and H1299 cells as previously described^28^. Briefly, ~10^6^ A549 or H1299 cells in 100mm dishes were pretreated with 40 μM T-96 or DMSO for 8 hours. Subsequently, the collected cells were washed once with chilled PBS and resuspended in 1.5 mL PBS containing a protease inhibitor cocktail. After being distributed into 12 tubes, the cells of each group were heat shocked in the Bio-Rad T100 thermal cycler at indicated temperatures for 3 minutes to denature proteins, and immediately cooled down to room temperature for another 3 minutes. Finally, the samples were subjected to three freeze-thaw cycles with liquid nitrogen and a thermal cycler set at 25 °C to lyse cells, and they were centrifuged at 15,000 g for 15 min at 4 °C to pellet cell debris along with precipitated and aggregated proteins. The supernatant was further analyzed by Western blot. The protein bands were quantified using ImageJ and data from three independent biological replicates were plotted.

### Protein extraction and western blotting

Cells were collected and lysed in RIPA buffer containing a protease and phosphatase inhibitor cocktail (1861280, Thermo Scientific) for 10 min on ice. The lysates were then centrifuged at 15000 g for 15 minutes at 4 °C to remove the insoluble materials. The protein concentrations were measured using the Enhanced BCA Protein Assay Kit (P0010, Beyotime Biotechnology, Shanghai, China). Then all the samples were heated at 95 °C for 10 min in 1X Sample Buffer (MB01015, GenScript). 30 μg proteins in each sample were separated by GenScript ExpressPlus™ PAGE Gels and transferred to polyvinylidene difluoride (PVDF) membranes (IPVH00010, Millipore). The membranes were blocked with 5% non-fat milk (232100, BD) and 0.1% Tween-20 at room temperature for 1 h and then incubated overnight at 4 °C with a 1:2000-dilution of Rabbit Anti-LRPPRC/GP130 antibody (ab97505, Abcam), a 1:1000-dilution of Total OXPHOS Rodent WB Antibody Cocktail produced in mouse (ab110413, Abcam), and a 1:10000-dilution Monoclonal Anti-β-Actin antibody produced in mouse (A5316, Sigma). After being washed by TBST (0.05% Tween20), the membranes were incubated with a 1:1000-dilution of HRP-labeled Goat Anti-Rabbit IgG(H+L) (A0208, Beyotime) or a 1:1000-dilution of HRP-labeled Goat Anti-Mouse IgG(H+L) (A0216, Beyotime) at room temperature for 1 h, respectively. The protein bands were visualized in TANON-5200MUTI using super enhanced chemiluminescence (ECL) detection reagent (P1050, Applygen, Beijing, China) and quantified using ImageJ software.

### RNA extraction and RT-qPCR analysis

The cells were collected and lysed with 1 mL TRIzol reagent (15596-026, Invitrogen Life Technologies). The RNA extraction was performed following the manufacturer's instructions for the TRIzol reagent. The reverse transcription was carried out using the GoScript™ Reverse Transcription System (A5000, Promega). Quantitative reverse transcription-polymerase chain reaction (RT-qPCR) analysis was performed using TB Green® Premix DimerEraser™ (Perfect Real Time) (RR091A, Takara) with the Applied Biosystems 7500 Fast Real-Time PCR System (Thermo Fisher Scientific). The data were analyzed following the 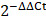 method and calculated using 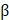-actin or RPLP0 as the internal control. Primers used in this study are shown in the Table S1.

### RNA Immunoprecipitates (RIP)

The RIP procedure was performed using a Magna RIP Kit (17-700, Millipore). A549 cell lines were lysed using RIP lysis buffer and subsequently subjected to immunoprecipitation with Anti-LRPPRC/GP130 antibody (ab97505, Abcam) against LRPPRC, utilizing protein A/G magnetic beads. Magnets were used to immobilize the complexes bound to magnetic beads while washing off the unbound materials. Subsequently, the remaining RNA was extracted. Total immunoprecipitated RNA from A549 cells was purified with the RNeasy MinElute Cleanup Kit (74204, Qiagen) according to the manufacturer's instructions.

### RNA-seq and data analysis

Total RNA samples were isolated from cells upon DMSO and 20 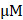 T-96 treatment for 24 h as described above. Total RNA samples that meet the following requirements were used in subsequent experiments: RNA integrity number (RIN) > 7.0 and a 28S:18S ratio > 1.8. RNA-seq libraries were generated and sequenced by CapitalBio Technology (Beijing, China). The NEB Next Ultra RNA Library Prep Kit for Illumina (NEB) was used to construct the libraries for sequencing. The final libraries were quantified using KAPA Library Quantification kit (KK4824, KAPA Biosystems, South Africa) and an Agilent 2100 Bioanalyzer. After RT-qPCR validation, libraries were subjected to paired-end sequencing with pair-end 150-base pair reading length on an Illumina NovaSeq sequencer (Illumina).

The genome of the human genome version of hg38 was used as the reference. The gene expression analyses were performed with StringTie (v1.3.3b). limma (v3.54.2) was used to analyze the DEGs (differentially expressed genes) between samples. Thousands of independent statistical hypothesis testing was conducted on DEGs, separately. Then a p-value was obtained, which was corrected by the FDR method. The corrected P-value (q-value) was calculated by correcting using the BH method. p-value or q-value were used to conduct significance analysis. Parameters for classifying significantly DEGs are ≥2-fold differences (|log2FC|≥1, FC: the fold change of expressions) in the transcript abundance and p ≤ 0.05. All genes were used to perform PCA. PCA results were visualized in a two-dimensional coordinate space according to two major principal components.

### Gene Ontology (GO) and Kyoto Encyclopedia of Genes and Genomes (KEGG) enrichment analysis

In this study, based on the Enrichr database, the clusterProfiler (v4.6.2), an R package, was used to analyze DEGs' GO and KEGG pathways to determine the most abundant biological pathways and functions associated with DEGs. p < 0.05 was the inclusion criterion.

### Gene Set Enrichment Analysis (GSEA)

GSEA was performed using GSEA desktop software (v4.1.0) from the Broad Institute. The reference gene set used in this study was c2.cp.kegg.v7.5.symbols.gmt, with nominal P < 0.05 and FDR < 5% used as the threshold to screen significant enrichment functions and pathways.

### Immunofluorescence assay

The A549 cells were treated with different concentrations of T-96 (20 μM, 10 μM, 0) for 48 h and 72 h. Then, Mito Tracker Deep Red FM (1:10000; M22426, Invitrogen) was incubated at 37 °C for 1 h. After being washed with DPBS, the cells were fixed with 4% paraformaldehyde for 15 min at room temperature and permeabilized with Triton X-100 for 10 min. Subsequently, the cells were washed again with DPBS and blocked with 1% BSA (PBST) at room temperature for 1 h. Following another round of washing with DPBS, nuclei were stained with Hoechst33258 (2.5 μg/mL; H21491, Invitrogen) at room temperature for 10 min. Finally, the cells were washed with DPBS for 15 min before being observed using a confocal fluorescence microscope (FV1000, Olympus, Tokyo, Japan).

### OCR seahorse assay

The A549 cells were harvested and suspended in DMEM medium at a concentration of 4×10^5^ cells/mL. Subsequently, 100 μL of the cell suspension was added to Seahorse 24-well XF Cell Culture microplate and cultured at 37 °C with 5% CO_2_ for adherence. Then the cells were treated with or without 5 μM T-96 in 250 μL medium for 72 hours. According to the manufacturer's instructions of Agilent Seahorse XFe24, the Seahorse XF Sensor Cartridge was hydrated and placed in a CO_2_-free cell incubator at 37 °C for 24 h the day before the following assay. After that, the cells were washed and cultured in 500 μL of pre-warmed Seahorse complete medium in a CO_2_-free cell incubator at 37 °C for 60 minutes. Then the oligomycin (final concentration 1.5 μM), carbonyl-cyanide-4-(trifluoromethoxy) phenyhydrazone (FCCP, final concentration 0.5 μM), and a mix of rotenone and antimycin A (final concentration 0.5 μM) were loaded into the injection ports of the hydrated sensor cartridge and added to the cultures in sequence. The addition of oligomycin was employed to evaluate oxidative leak. The administration of FCCP could induce maximal stimulation of mitochondrial electron transport. Rotenone and antimycin A were used to measure extra-mitochondrial respiration.

### Molecular docking

Molecular docking was performed between protein LRPPRC with T-96. The prediction 3D structure of LRPPRC (AlphaFold ID: AF-P42704-F1) was downloaded from the AlphaFold protein structure database (https://alphafold.ebi.ac.uk/). LRPPRC was imported into AutoDockTools 1.5.7 for hydrogenation, charge calculation, and non-polar hydrogen combination, and then the result was saved in PDBQT format. Set the size of the Grid Box to 40 × 40 × 40. Finally, AutoDock4 was used for molecular docking, and PyMOL was used to visualize the results.

### Animal models

4-6 weeks-old male nude mice (BALB/c-Nu) and sterilized food were purchased from Hangzhou Medical College. 1 × 10^7^ A549 cells in 200 μL PBS were injected into nude mice subcutaneously and the mice were randomly divided into two groups. Demethylzeylasteral were dissolved in 20% cyclodextrin to a final concentration of 1 mg/mL, and each mouse received a dose of 100 μL by intraperitoneal injection every two days, and 20% cyclodextrin was utilized as a control placebo. The mice were sacrificed depending on the tumor size and the level of animal discomfort. The xenograft tumors were fixed with 10% formalin and subjected to further immunohistochemistry analysis. The study adhered to the principles of the 3Rs (Replacement, Reduction, and Refinement) to ensure the welfare of the animals involved. Efforts were made to minimize pain and distress, and all procedures were performed under appropriate anesthesia. Humane endpoints were established to guide the decision for euthanasia when necessary. The researchers are committed to the ethical treatment of animals in research and have ensured that all aspects of the study meet the highest standards of animal care.

### Immunohistochemistry (IHC)

Tissues were embedded with paraffin and then cut into sections with a thickness of 5 μm. After deparaffinization, hydration, blocking, antibodies (Anti-LRPPRC, Abcam, ab97505, 1:1000; Anti-Ki67, Proteintech, 27309-1-AP, 1:500) incubating, and DAB staining, the staining pictures of tissue sections were scanned by a Pathology workstation (Olympus, Japan).

### Statistical analysis

All values were presented as mean
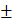
SD or mean
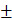
SEM unless otherwise specified. Differences were analyzed by one-way ANOVA test or two-way ANOVA test in GraphPad Prism 9; significance was defined as p < 0.05. * P < 0.05, ** *P* < 0.01, *** P < 0.001, **** P < 0.0001.

## Results

### T-96 directly binds to LRPPRC

**Figure 1:**
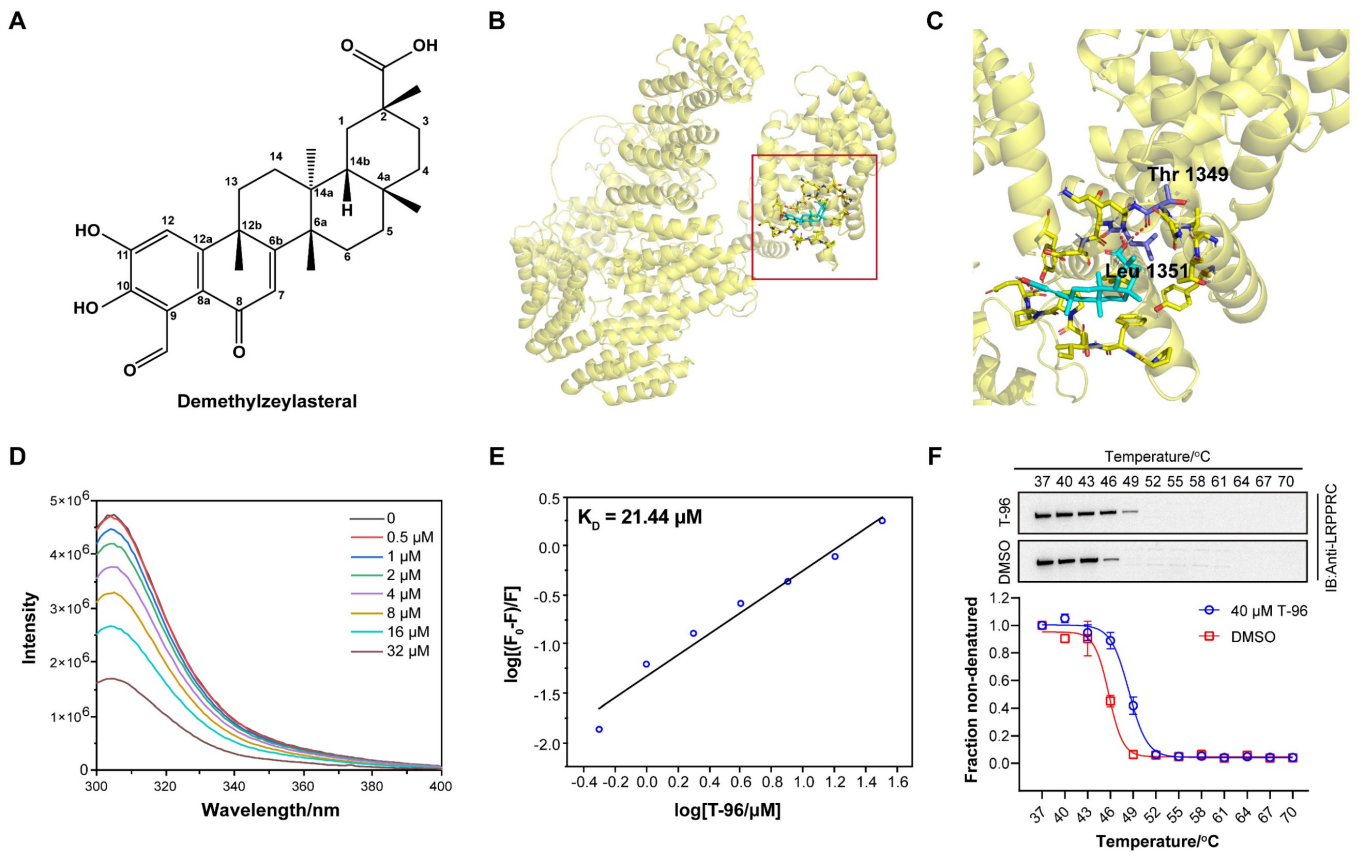
** T-96 directly binds to LRPPRC. (A)** Chemical structure of T-96. **(B)** The predicted global binding mode of T-96 to LRPPRC by docking analysis. **(C)** The docking site is shown in an enlarged view. Cyan represents T-96, purple represents amino acid residues, and yellow represents LRPPRC. **(D)** Fluorescence emission spectra of LRPPRC∆5 with the addition of T-96, λ_ex_ = 280 nm, λ_em_ = 305 nm. **(E)** Modified Stern-Volmer plot for fluorescence quenching of LRPPRC∆5 (1121aa-1394aa) in the presence of T-96. **(F)** Western blot analysis and thermal shift curves of LRPPRC from CETSA in A549 pretreated with 40 μM T-96 (mean ± s.e.m.; n = 3 biological replicates).

In previous work, we developed an aptamer-assisted high-throughput method based on competitive fluorescence polarization assay and screened out the traditional Chinese medicine monomer T-96, which disrupted the LRPPRC's aptamer binding ability^22^ (Figure 1A). To investigate if LRPPRC is a target of T-96, we first carried out the molecular docking of protein LRPPRC with ligand T-96 using AutoDock 4.2.6. According to the prediction results, the estimated lowest free energy of binding between T-96 and LRPPRC is -7.38 kcal/mol, which indicates that the interaction is relatively robust^29^. Furthermore, the amino acid analysis indicated that it is the amino groups of Thr1349 and Leu1351 that might mediate the interaction of T-96 and LRPPRC (Figure 1B, 1C, S1, and S2).

Next, we purified C terminals of LRPPRC (1121aa-1394aa, LRPPRC∆5) and applied fluorescence titration assay and biolayer interferometry (BLI) assay to test its interaction with T-96. Fluorescence titration assay showed T-96 interacted with the RNA binding domain of LRPPRC with the equilibrium dissociation constants (K_D_) of 21.44 μM (Figure 1D-E). Similar binding strength was also confirmed by the BLI assay, which showed a K_D_ value of 20.55 μM (Figure S3A). These results indicated that T-96 directly bound to the RNA binding domain of LRPPRC *in vitro*.

Additionally, the cellular thermal shift assay (CETSA) by using A549 and H1299 cells further confirmed that T-96 bound to LRPPRC in the complicated cellular environment. Upon ligand binding, the thermal stability of the protein was enhanced, resulting in a positive shift in the CETSA melting curve. Western blotting analysis showed the presence of LRPPRC at the lower test temperatures followed by its disappearance with increased temperature in the control groups. However, the addition of T-96 resulted in a strong band of LRPPRC at 49 °C (Figure 1F), which suggested that T-96 was effectively engaged by the LRPPRC protein in both A549 (Figure 1F) and H1299 cells (Figure S3B). The above experimental results confirmed that T-96 directly bound to the RNA binding domain of LRPPRC, which could also inhibit the nucleic acid binding ability of LRPPRC.

### T-96 inhibits the biogenesis of the oxidative phosphorylation complex

LRPPRC is primarily located in mitochondria and is responsible for the maturation and stability of mt-mRNA by directly binding with them^30^, ^31^. All 13 proteins that encode mt-mRNA are essential for the synthesis of OXPHOS complexes. Suppression of LRPPRC could cause mt-mRNA to become unstable, leading to impairments in the synthesis of OXPHOS complexes^32^. As T-96 directly bound to LRPPRC, we performed transcriptome analyses of A549 cells to determine whether T-96 affects the oxidative phosphorylation pathway in tumor cells.

**Figure 2:**
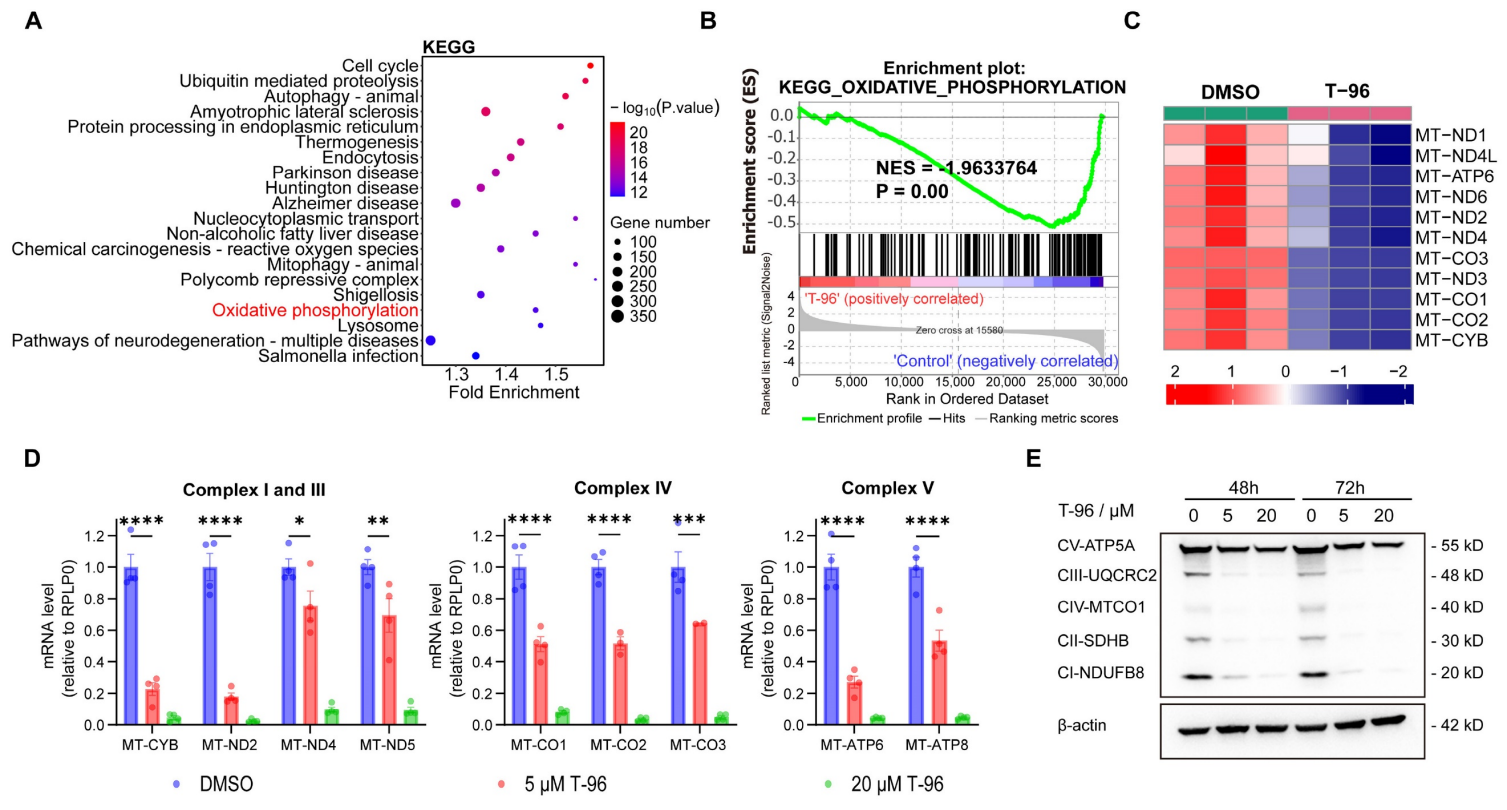
**Effect of T-96 treatment on the biogenesis of oxidative phosphorylation system. (A)** The top 20 KEGG pathway terms enriched with a P value < 0.001 were significantly regulated by T-96. **(B)** GSEA results of KEGG oxidative phosphorylation set in A549 cells treated by T-96. **(C)** Heatmap of the differentially expressed mitochondrial coding genes in A549 cells with and without T-96 treatment. Red stripes represent high-expression genes. Blue stripes represent low-expression genes.** (D)** mt-mRNA levels of the complex I, III, IV, and V subunits in A549 cells (mean ± s.e.m.; n = 4 biological replicates).** (E)** Western blot analyses of OXPHOS complex subunits in A549 cells pretreated with different T-96 concentrations.

Indeed, principal-component analysis (PCA) of transcriptome data from the T-96 treated groups and control groups showed a shift in principal component 1 (PC1) and PC2, indicating a significant influence of T-96 treatment on the gene expression profile (Figure S4A and S4B). To gain insight into the signaling and metabolism pathways involved in these differentially expressed genes (DEGs), functional enrichment analyses were performed based on Gene Ontology (GO) and the Kyoto Encyclopedia of Genes and Genomes (KEGG) database. As expected, KEGG analysis revealed that the oxidative phosphorylation pathway was significantly enriched among the top 20 pathways satisfying P.value < 0.001 (Figure 2A). This tendency was also supported by the GO enrichment results (Figure S4C). Gene Set Enrichment Analysis (GSEA) further demonstrated that the oxidative phosphorylation pathway was negatively correlated with T-96 treatment (Figure 2B). Additionally, a heatmap of the mitochondrial coding genes generated from transcriptome data showed that 12 of all the 13 genes were significantly downregulated (Figure 2C). Moreover, we obtained the RNA sequencing data of hepatoma cells before and after T-96 treatment (PRJNA763373) and performed the enrichment analysis. The results from this public dataset also indicated that T-96 treatment led to downregulation of mt-mRNA expression, thereby suppressing OXPHOS pathway (Figure S5).

The inhibition effect of T-96 on the OXPHOS gene set was further validated through RT-qPCR and Western blot (Figure 2D and E). Accordingly, RT-qPCR analysis revealed a remarkable dose-dependent reduction in the levels of mt-mRNAs following T-96 administration. Specifically, MT-ND2 of complex I and MT-CYB of complex III exhibited an approximately 80% decrease in mRNA level upon treatment with 5 μM T-96. Additionally, we observed a significant dose-dependent decrease in the levels of subunits (NDUFB8, UQCRC2, and MT-CO1) of respiratory chain complexes I, III, and IV. Furthermore, there was a decrease in the levels of subunits ATP5A in complexes V, whose stability relies on subunits translated by mt-mRNAs. These results were consistent with the transcriptome data and demonstrated that T-96 could inhibit the biogenesis OXPHOS complex by suppressing the expression of proteins encoded by mtDNA.

### T-96 suppresses the generation of the OXPHOS complexes by targeting LRPPRC

**Figure 3:**
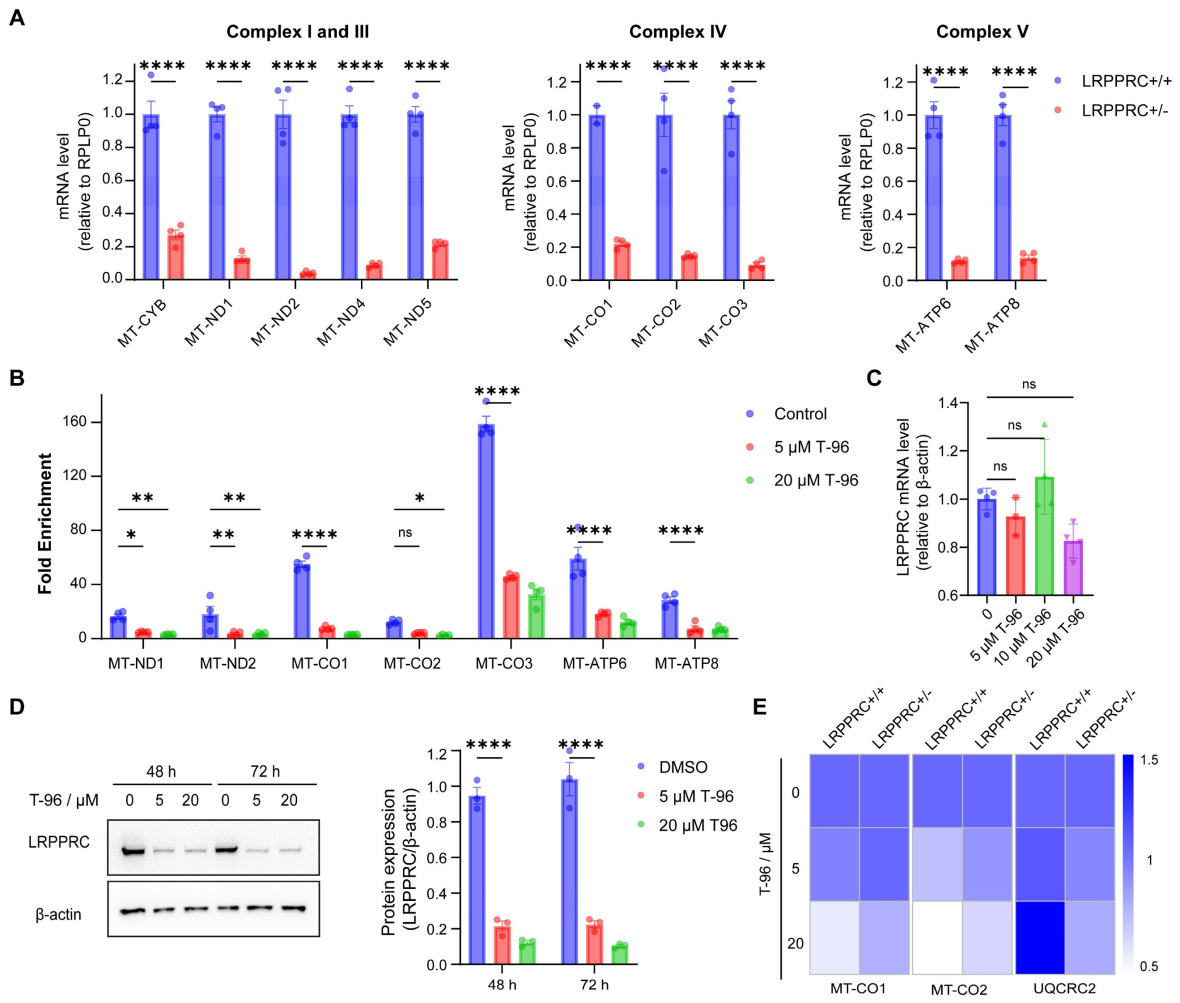
**T-96 suppresses the OXPHOS complexe generation by targeting LRPPRC. (A)** mt-mRNA levels of the complex I, III, IV, and V subunits before and after LRPPRC knockout in A549 cells (mean ± s.e.m.; n = 4 biological replicates). **(B)** mRNA levels of mitochondrial transcripts enriched by LRPPRC antibody-based RIP assay (mean ± s.e.m.; n = 4 biological replicates). **(C)** mRNA level of LRPPRC in A549 cells treated with different concentrations of T-96.** (D)** Western blot analysis of LRPPRC in A549 cells treated with different concentrations of T-96 for 48 hours and 72 hours. **(E)** mRNA levels of MT-CO1, MT-CO2, and UQCRC2 in A549-wild type and A549-LRPPRC+/- cells pretreated with different concentrations of T-96 (mean ± s.e.m.; n = 4 biological replicates).

According to the above experimental results, T-96 could suppress the generation of the OXPHOS complexes and also directly bind to LRPPRC. To investigate whether T-96 inhibits the expression of the OXPHOS complex through LRPPRC, we first checked the effect of LRPPRC on the expression of OXPHOS complex subunits. Consistent with previous research^33^, knocking down LRPPRC in A549 cells led to a significant reduction in the mRNA level of OXPHOS complex subunits as shown in Figure 3A, which confirmed the function of LRPPRC in maintaining OXPHOS.

As T-96 directly bound to LRPPRC and LRPPRC bound to mt-mRNAs, we performed the RNA Binding Protein Immunoprecipitation (RIP) experiment to analyze the antagonistic effect of T-96 on the mt-mRNA's binding ability of LRPPRC. LRPPRC-specific antibody was used to enrich mRNAs that were bound to LRPPRC, and the enriched RNAs were further purified for PCR quantification. The analysis showed that mt-mRNAs enriched by LRPPRC antibody significantly decreased after T-96 treatment. Notably, 5 μM T-96 reduced the LRPPRC-binding mitochondrial transcripts by about 70% (Figure 3B), such as MT-CO2, MT-ND1, and so on. Thus, T-96 weakened the mt-mRNAs' binding ability of LRPPRC.

As the interaction between LRPPRC protein and mt-mRNA also helps to maintain the stability of LRPPRC itself, depriving mt-mRNAs can induce the degradation of LRPPRC protein. Immunoblotting showed that T-96 significantly reduced the protein level of LRPPRC. In A549 cells, 20 μM T-96 reduced 90% of the LRPPRC (Figure 3D). However, there was no statistically significant difference in the effect of T-96 on the mRNA level of LRPPRC (Figure 3C), suggesting that T-96 degraded the protein LRPPRC without affecting its transcription. Together, T-96 acted a specific bi-functional inhibitor of LRPPRC in mitochondria which inhibited the mt-mRNA binding ability of LRPPRC and also induced its degradation, then further affected the provision of new OXPHOS complex subunits.

To further validate the T-96-induced mt-mRNA inhibition is LRPPRC-dependent, T-96 treatment was carried out on both LRPPRC knockout cells and parent cells. In parent cells, 20 μM T-96 reduced mitochondrial transcripts by 80%, while in LRPPRC knockout cells, the inhibitory effect of T-96 on mt-mRNA level was not significant (Figure 3E). Moreover, Western blot results confirmed that the inhibitory effect of T-96 on OXPHOS-related protein levels was reduced, and the reduction of OXPHOS-related protein levels in LRPPRC-deficient cells induced by T-96 was not significant compared to that in A549 parent cells (Figure S6A, C and D). This indicated that the inhibitory effect of T-96 on mt-mRNA and protein was diminished when LRPPRC expression was reduced. Additionally, we conducted a comparative analysis of the effects of T-96 between A549 cells (high LRPPRC expression) and H1688 cells (low LRPPRC expression) on OXPHOS expression. The results showed that T-96 exhibited a weaker effect on the expression of OXPHOS protein in H1688 cells, and no clear dose-response correlation was observed at the RNA levels (Figure S6B, C, and E). Therefore, it can be concluded that the suppression of OXPHOS complex subunits' expression by T-96 was dependent on LRPPRC.

### T-96 induces mitochondrial dysfunction

It is known that OXPHOS complexes generate a pH difference between the matrix and intermembrane spaces of mitochondria, and this proton gradient is essential for mitochondria oxygen consumption and ATP synthesis^34^. Considering that T-96 can suppress the expression of OXPHOS complexes, we then investigated the effect of T-96 on mitochondria function in tumor cells.

**Figure 4:**
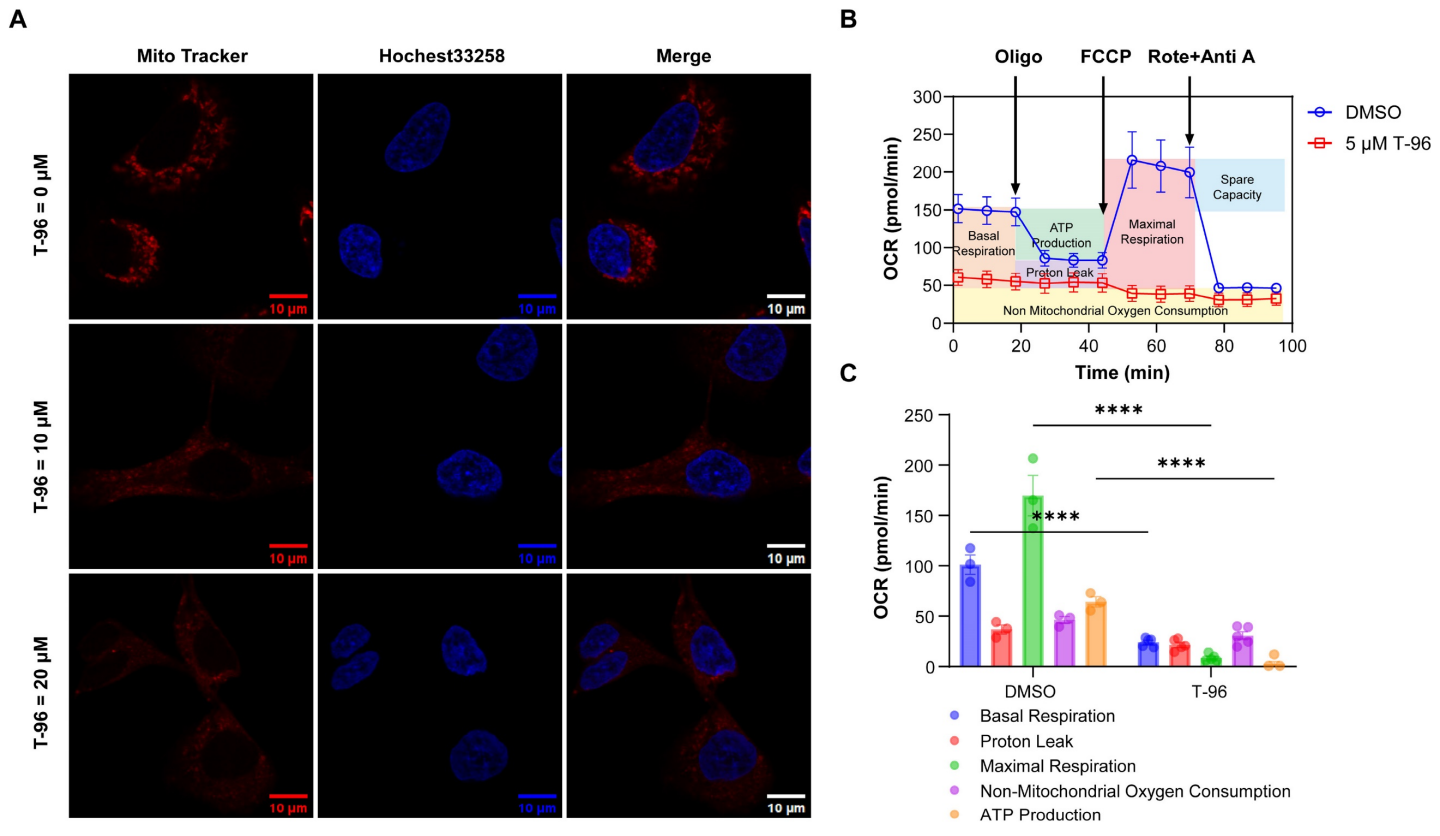
** T-96-induced LRPPRC inhibition leads to mitochondrial dysfunction. (A)** Confocal imaging of mitochondria (Red), and nucleus (Blue) in A549 cells treated with different concentrations of T-96 for 48 hours. **(B)** Profile of OCR in A549 cells tested by Seahorse analyzer. Cells were pretreated with T-96 (5 μM, 72 hours) or the control solution (DMSO). After the mitochondrial stress test started, oligomycin (20 min later), FCCP (48 min later), and a mixture of rotenone and antimycin A (70 min later) were added in sequence (mean ± s.e.m.; n = 3-5). **(C)** Quantification of basal respiration, proton leak, maximal respiration, non-mitochondrial oxygen consumption, and MT-ATP production for A549 cells pretreated with 5 μM T-96 or DMSO control (mean ± s.e.m.; n = 3-5).

The mitochondrial proton gradient was first tested through a staining assay with a mitochondrial tracker dye, and the results showed that cells treated with T-96 could not be stained as effectively as the control group by this dye. As the effectiveness of the mitochondrial tracker dye relies on the presence of a functional mitochondrial proton gradient, these results indicated an impaired mitochondrial proton gradient caused by T-96 (Figure 4A).

A cell mitochondrial stress test was next performed to measure oxygen consumption rate (OCR) and ATP production. In this assay, four inhibitors against mitochondrial respiration, oligomycin, FCCP, and antimycin A/rotenone, were added to the cells in order, and the OCR profile for A549 cells was presented in Figure 4B. In contrast to the control cells, the T-96 treated cells showed a significantly decreased basal respiration only slightly higher than non-mitochondrial oxygen consumption. No significant effects of these inhibitors on respiration were observed, indicating a severe dysfunction of OXPHOS complexes. Note that the low maximum respiration of T-96 treatment cells also implied a disability to respond to more energy demand challenges. Further data analysis proved that T-96 resulted in a significant decrease in basal OCR and mitochondrial ATP production (Figure 4C). Taken together, T-96 could suppress the mitochondria function of A549 cells, including proton gradient formation, oxygen consumption, and energy production.

### T-96 inhibits LUAD cells' malignant phenotype *in vitro* and inhibit subcutaneous tumorigenesis *in vivo*

**Figure 5:**
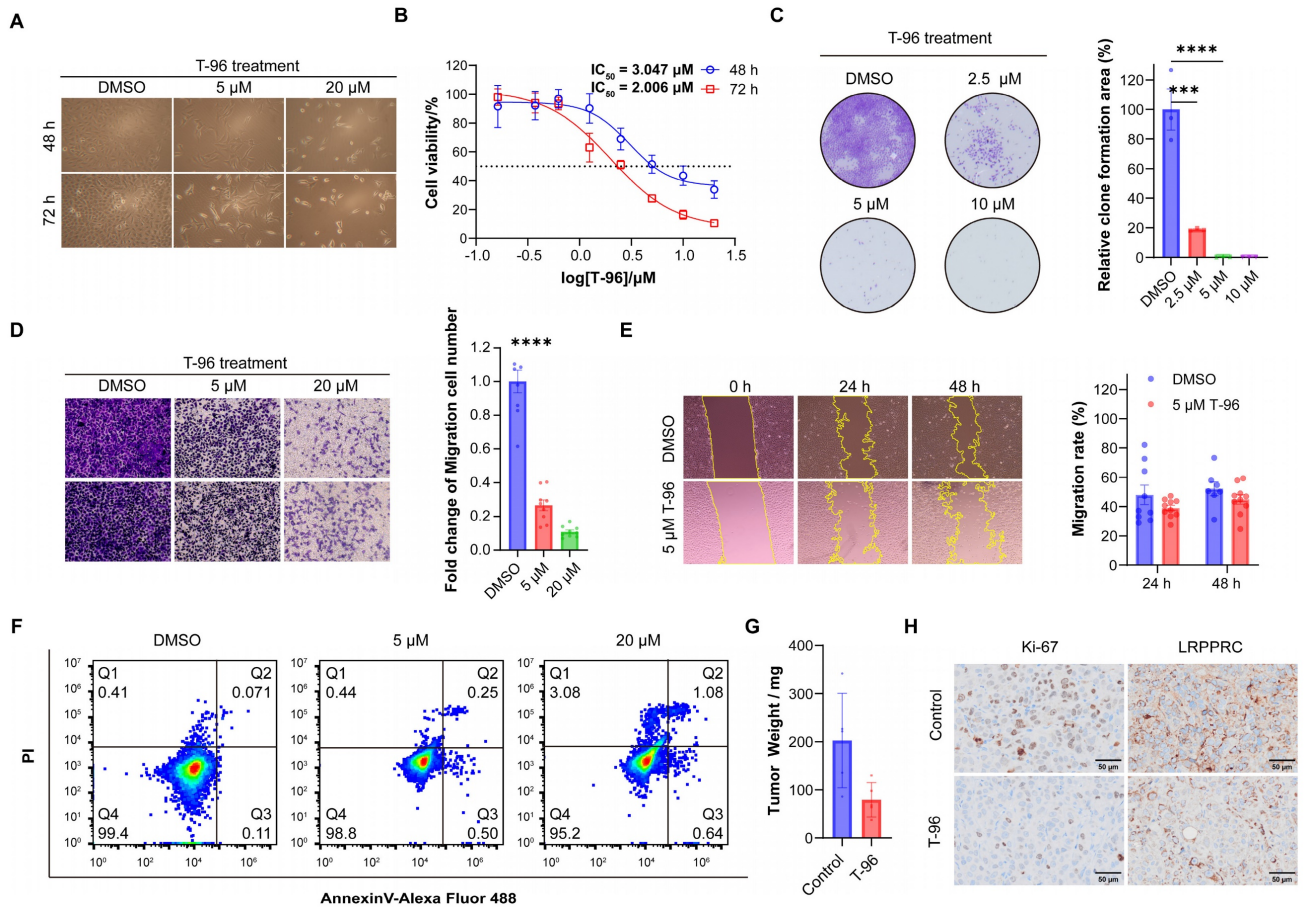
**T-96 inhibits tumor malignant phenotype in a non-apoptotic manner and inhibits the growth of A549 cells *in vivo*. (A)** Cell morphology imaging of A549 cells treated with different concentrations of T-96. **(B)** Effects of T-96 on the viability of A549 cells were evaluated by 48 h and 72 h CCK8 assays. **(C)** The results of colony‑formation assays with A549 cells treated with different concentrations of T-96. **(D)** Transwell invasion assay results of A549 cells after T-96 treatment. **(E)** Scratch wound healing assay results of A549 cells after T-96 treatment. **(F)** Apoptosis in A549 cells was analyzed following 96 hours of T-96 treatment using flow cytometry. Cells were stained with AnnexinV-Alexa Fluor 488 and PI. Results are representative of three independent experiments. Q4, double-negative (Annexin V-Alexa Fluor 488 and PI negative) represented viable cells; Q3, Annexin V-Alexa Fluor 488 positive and PI negative represented apoptotic cells; Q2, Annexin V-Alexa Fluor 488 & PI double-positive represented necrotic cells. **(G)** Tumor weight of subcutaneous A549 cells treated with indicated drugs (mean ± s.e.m.).** (H)** IHC images of indicated proteins in A549 cells xenografts treated with T-96.

As LRPPRC is an emerging protein biomarker and a promising therapeutic target for the most lethal PP subtype of LUAD^22^, we hypothesized that T-96 could be a candidate drug for the treatment of lung adenocarcinoma. To confirm this hypothesis, we examined the effect of T-96 on LUAD cells. We found that T-96 significantly caused cell growth inhibition and morphological changes in A549 cells. Most of the cells became round and presented a dead cell morphology after 48 and 72 hours of incubation with 5 μM T-96 (Figure 5A). The half-maximal inhibitory concentration (IC_50_) of T-96 at 48 h and 72 h with A549 cells were further tested to be 3.0 μM and 2.0 μM, respectively (Figure 5B). Additionally, the colony formation assay showed that T-96 markedly declined the colony size and number, indicating the suppression of T-96 on colony formation ability in A549 cells (Figure 5C). All these results demonstrated that T-96 has a notable capacity to inhibit the growth and proliferation of A549 cell line. Besides, transwell and invasion assays showed that the number of cells penetrating through wells dramatically reduced after T-96 treatment, and exhibited that T-96 could strongly repress the invasion abilities of A549 cells (Figure 5D).

To assess the effects of T-96 on A549 cell migration ability, wound healing assays were carried out, and the results presented that T-96 significantly decreased the rate of wound healing (Figure 5E), which elucidated the inhibition of T-96 on the migration of A549 cells. In addition, all these inhibitions on migration and invasion were concentration-dependent. We also investigated whether the inhibition of malignant phenotype induced by T-96 is related to the inhibition of LRPPRC protein. Following LRPPRC knockdown, we compared the sensitivity of cells to T-96 treatment. The results showed that the IC_50_ of T-96 for A549 cells was increased and the morphology changes induced by T-96 became slight (Figure S7). Therefore, T-96 can inhibit the malignant phenotype of cancer cells, and this inhibitory effect is dependent on the expression of the LRPPRC protein.

Previous studies have shown that T-96 can cause apoptosis in prostate cancer^35^ and melanoma^36^. To determine whether T-96 suppressed A549 cell proliferation by activating apoptosis, we conducted flow cytometry assays after exposing the cells to T-96. As indicated in Figure 5F and S8, T-96 did not induce cell apoptosis in A549 cells even after being treated with 20 μM for 96 hours. Taken together, these data demonstrated that T-96 inhibited LUAD cell proliferation in a non-apoptotic manner.

To further investigate the inhibitory effect of T-96 on LUAD cells *in vivo*, A549 cells were used to establish a nude mouse xenograft model for *in vivo* experiments (Figure S9A). The nude mice were randomly assigned to two groups: the control group and the T-96 treatment group. All the mice were sacrificed after treatment, and the xenograft tumors were removed and weighed. The tumor proliferation curve showed T-96 administration could reduce the growth rate of subcutaneous tumors. Furthermore, the statistical analysis of tumor weight demonstrated that mice injected with T-96 had smaller tumor weights compared to those injected with placebo (Figure 5G, S9B and S9C). Additionally, we prepared tumor tissue sections from the A549 cell subcutaneous xenograft and performed immunohistochemistry assay (IHC). The results showed that the expression level of Ki-67 in the T-96 injection group was significantly lower than that in the control group, indicating that T-96 inhibited cell proliferation, which is consistent with the findings reported by other groups^37^, ^38^. Furthermore, the expression level of LRPPRC was also reduced in the T-96 injection group compared to the control group (Figure 5H, S10, and S11). These findings suggested that T-96 could also downregulate LRPPRC expression and suppress the proliferation of LUAD cells *in vivo*.

## Discussion

In the past decades, Chinese herbal medicine has been recognized worldwide as a rich source for the discovery of novel drugs. The search for new antitumor drugs from natural sources is one of the most important approaches to cancer prevention and therapy. Many traditional Chinese medicine monomers have unique anti-cancer beneficial features. Previous studies have shown that T-96, extracted from *Tripterygium wilfordii* Hook. f., exhibits antitumor effects in various tumors. However, the specific binding target of T-96 has not yet been identified. In this work, we revealed that T-96 directly bound to LRPPRC, and worked as a specific bifunctional inhibitor of LRPPRC in mitochondria. In addition, we have discovered a novel pathway through which T-96 can inhibit tumors by suppressing the expression of OXPHOS complexes. Importantly, T-96 was the first traditional Chinese medicine monomer that could inhibit OXPHOS complex generation. Our study provided useful insights into the molecular mechanisms underlying the antitumor activity of T-96.

It has been reported that the reduction of LRPPRC expression is implicated in many human diseases, such as Alzheimer's disease and Parkinson's disease^39^-^42^, and the KEGG analysis also identified these pathways which were affected significantly by T-96 in this study (Figure 2A). Besides, many other pathways, including the cell cycle pathway, chemical carcinogenesis-reactive oxygen species, and p53 signaling pathway, were enriched in both the T-96 treatment group and the LRPPRC knockout group (Table. S2 and S3). These data not only demonstrated the inhibitory effect of T-96 on LRPPRC but also implied the sophisticated cross-regulation between these signaling pathways. It was also found that some signaling pathways were enriched in the T-96 treatment group but not in the LRPPRC knockdown group. This phenomenon may be caused by other targets of T-96 except for LRPPRC. Therefore, more detailed mechanistic studies of T-96 are necessary to fill in the gaps in the pharmacological mechanisms of T-96.

Recent works have clarified the indispensable role of mitochondrial OXPHOS in the occurrence and development of tumors. As OXPHOS is also an essential cellular process for normal cells, directly blocking the activity of OXPHOS complexes has been viewed as an unfeasible therapeutic strategy. Our work has proposed a new target, LRPPRC, for the antitumor therapeutic approach that only suppressed the generation of new OXPHOS complexes. Small molecules targeting LRPPRC would have a negligible effect on normal cells with low LRPPRC expression. Compared with the reported other two targets of OXPHOS, mitochondrial ribosome^21^ and POLRMT^16^, ^19^, LRPPRC presents unique advantages: universal high expression in multiple cancers, positive correlation with poor prognosis, and the potential broad applicability to tumor treatment (Figure S12). Currently, only two specific small molecule inhibitors of LRPPRC, T-96 and GAA, have been identified with K_D_ around 21 μM and 16.5 μM respectively^22^. Thus, it is requisite to discover more small molecule inhibitors of LRPPRC for the emerging therapy aimed at inhibiting mitochondrion biogenesis. Structural modification based on the two molecules is also a promising direction to improve the affinity and specificity in the future.

## Supplementary Material

Supplementary figures and tables 1-2.

Supplementary table 3.

## Abbreviations

ATP: adenosine triphosphate
DMSO: Dimethyl sulfoxide
LRPPRC: Leucine-rich pentatricopeptide repeat-containing protein
LUAD: Lung adenocarcinoma
mtDNA: mitochondrial DNA
mt-mRNA: mtDNA transcribed mRNA
OCR: oxygen consumption rate
OXPHOS: mitochondrial oxidative phosphorylation
POLRMT: RNA polymerase mitochondrial
TCM: Traditional Chinese Medicine
