# Supplementary material for: Demethylzeylasteral inhibits oxidative phosphorylation complex biogenesis by targeting LRPPRC in lung cancer
Source: J Cancer. 2025 Jan 1;16(1):227–40. doi: 10.7150/jca.92797 (PMC11660126; doi:10.7150/jca.92797)
Supplement: Supplementary file 1 — Supplementary figures and tables 1-2. [file jcav16p0227s1.pdf]

Supplementary information

Demethylzeylasteral inhibits oxidative phosphorylation complex biogenesis by targeting LRPPRC in lung cancer

Lina Wang[1,2], Wei Zhou[3], Wenxi Wang[3], Yuxin Liang[1,2], Qiqi Xue[3], Zhen Zhang[1], Jinghe Yuan[1], Xiaohong Fang[1,2,3]✉

[1] Key Laboratory of Molecular Nanostructure and Nanotechnology, CAS Research/Education Center for Excellence in Molecular Sciences, Institute of Chemistry, Chinese Academy of Science, Beijing 100190, PR China.

[2] University of the Chinese Academy of Sciences (UCAS), Beijing 100049, PR China.

[3] Hangzhou Institute of Medicine (HIM), University of Chinese Academy of Sciences (Zhejiang Cancer Hospital), Chinese Academy of Sciences, Hangzhou, Zhejiang 310022, PR China.

✉Corresponding authors: Xiaohong Fang, Key Laboratory of Molecular Nanostructure and Nanotechnology, CAS Research/Education Center for Excellence in Molecular Sciences, Institute of Chemistry, Chinese Academy of Science, Beijing 100190, PR China. Email: xfang@iccas.ac.cn

Table of Contents:

1. **SUPPORTING FIGURES**
2. **SUPPORTING TABLES**

SUPPORTING FIGURES

Figure S1

Evaluating AlphaFold2's predicted structures of LRPPRC

The predicted local distance difference test (pLDDT) is a per-residue measure of local confidence. It is scaled from 0 to 100, with higher scores indicating higher confidence and usually a more accurate prediction. A pLDDT above 90 would be taken as the highest accuracy category, in which both the backbone and side chains are typically predicted with high accuracy. In contrast, a pLDDT above 70 usually corresponds to a correct backbone prediction with misplacement of some side chains. Some regions below 50 pLDDT may be unstructured in isolation. The prediction results show that most of the pLDDT is greater than 90, and the average pLDDT is 77.57, indicating that the predicted LRPPRC structure has high local confidence.

Predicted aligned error (PAE) serves as an indicator of confidence, reflecting the expected deviation between the predicted and true positions of residues when the two structures are optimally aligned. This deviation is expressed in angstroms (Å), a unit of length at the scale of atoms and molecules. A lower PAE value signifies a higher degree of confidence in the model, suggesting that the predicted structure closely mirrors the actual protein structure. AlphaFold2 uses a color-coded system to represent PAE values across the protein sequence. Dark green tiles in the PAE plot correspond to regions where the model predicts the structure with high accuracy and low error, indicating that the positions of the residues are well-defined and reliable. Conversely, light green tiles denote areas where the prediction is less certain, with higher PAE values suggesting greater potential error in the structural model. In the case of the LRPPRC protein, AlphaFold2 exhibits high confidence in the structure spanning certain regions, specifically residues 77-649, 647-1011, and 1022-1394. These regions are marked by dark green tiles, signifying that the arrangement of residues within these ranges is considered to be predicted with a high degree of precision. However, the relative positions of these three are less certain. The lighter coloring of this region visually conveys AlphaFold's hesitation in predicting its exact arrangement.

In general, the predicted structure of LRPPRC exhibits high local confidence and accurately represents the internal arrangement of the three residues; however, there is limited certainty regarding the relative positions between these residues.

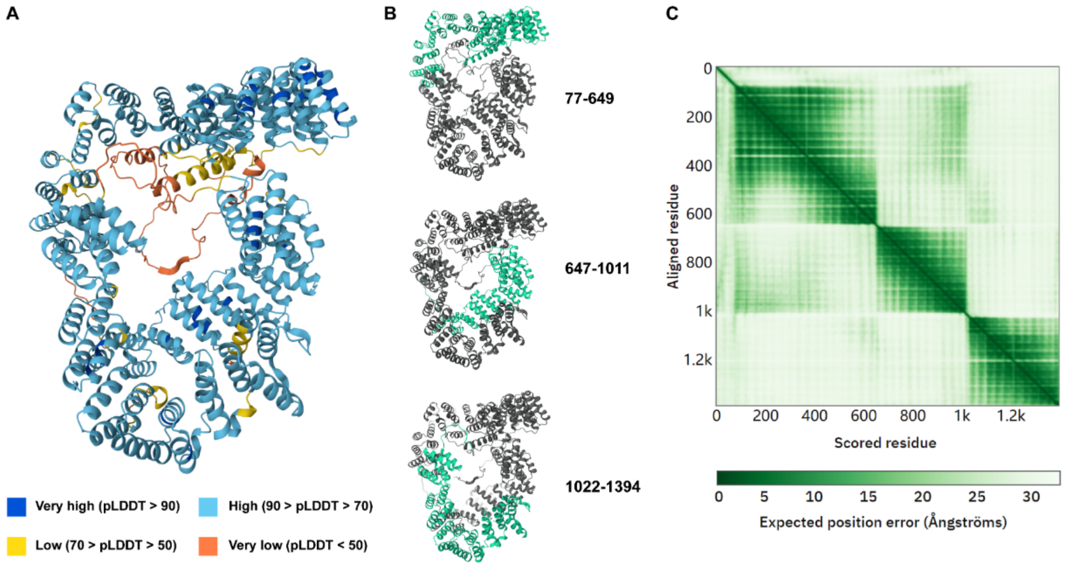

Figure S1. Evaluating AlphaFold2's predicted structures of LRPPRC (AlphaFold ID: AF-P42704-F1) using confidence scores. (A) AlphaFold produces a per-residue model confidence score (pLDDT) between 0 and 100. Some regions below 50 pLDDT may be unstructured in isolation. **(B)** The relative positions of residues (Green). **(C)** PAE (Predicted Aligned Error) plot show the regions of high confidence (dark green) and low confidence (pale green).

Figure S2

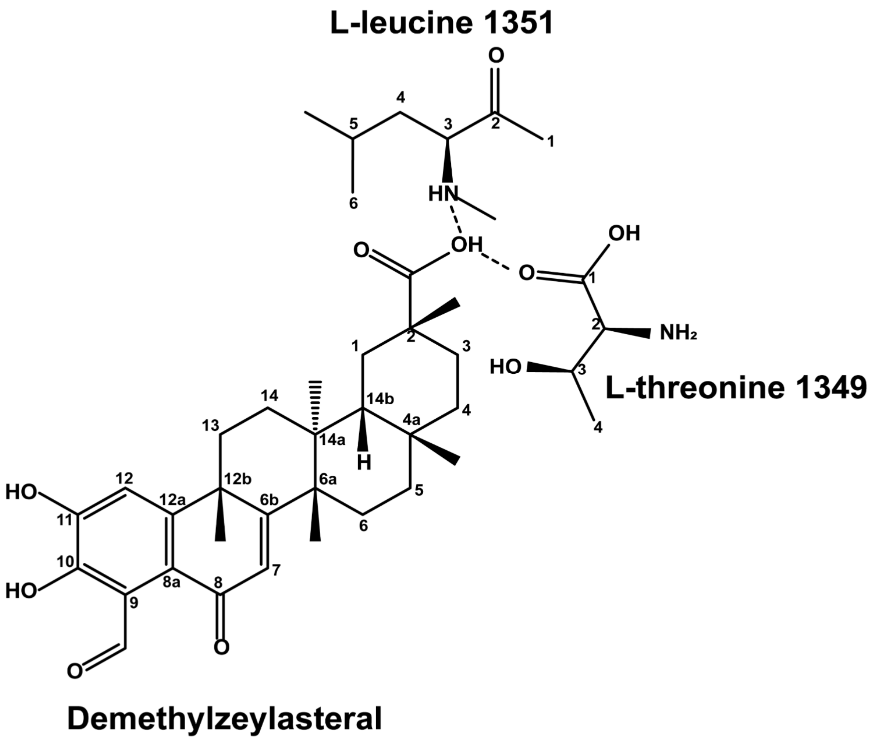

Figure S2. The molecular formula of the predicted binding site for T-96 to LRPPRC.

Figure S3

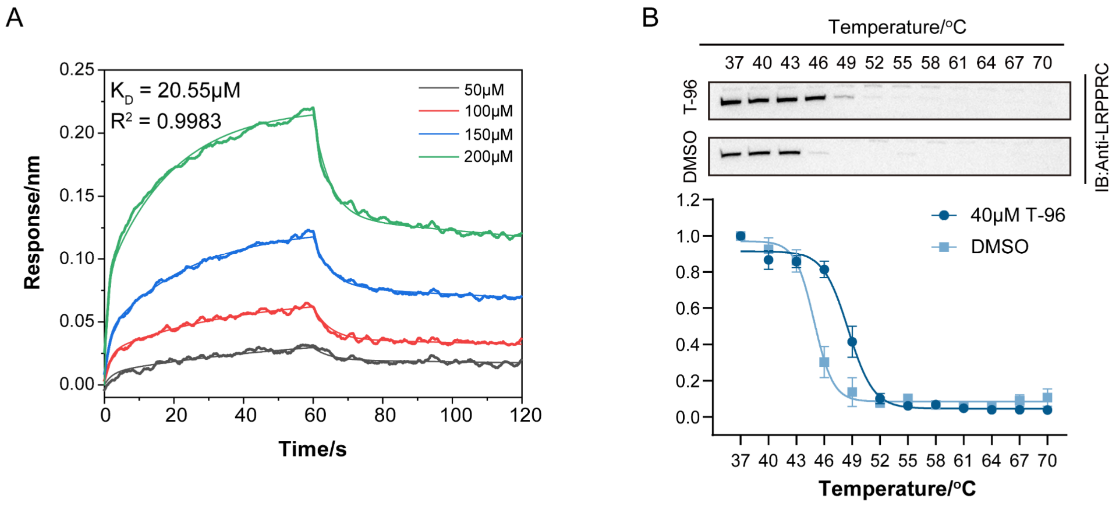

Figure S3. (A) T96-LRPPRCΔ5 biolayer interferometry sensorgram (2:1 model, R^2=0.9983). **(B)** Western blot analysis (upper panel) and thermal shift curves (lower panel) of LRPPRC from CETSA in H1299 cells pretreated with 40 μM T-96 (mean±s.e.m.; n=3 biological replicates).

Figure S4

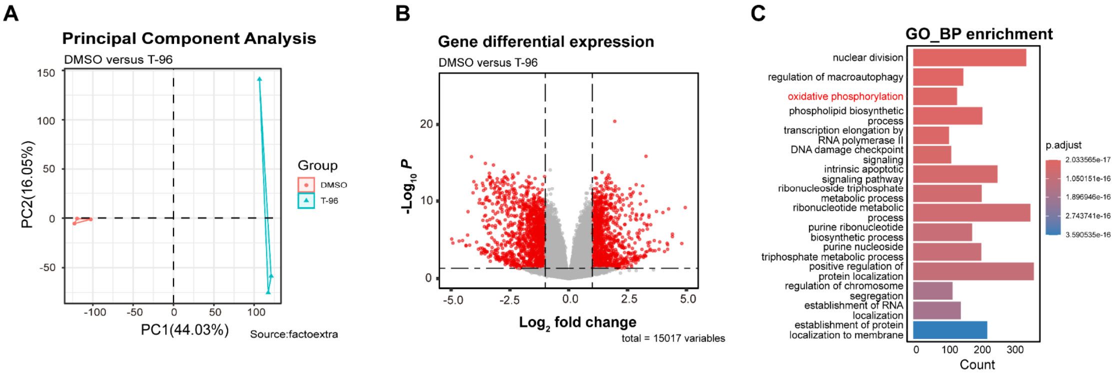

Figure S4. (A) PCA of transcriptomes data with and without T-96 treatment presented in a two-dimensional pattern**. (B)** Volcano plot map of the differentially expressed genes in T-96-treated A549 cells. **(C)** GO analysis for the significantly differentially expressed genes by T-96 compared to control in A549 cells.

Figure S5

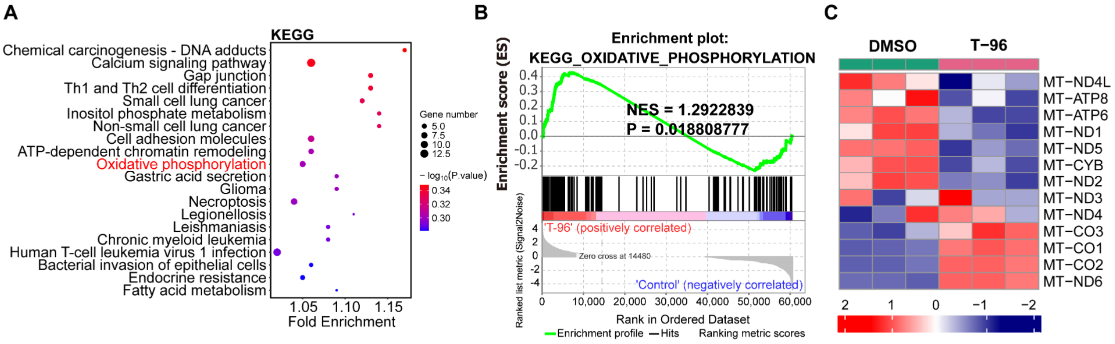

Figure S5. (A) KEGG analysis of the significantly differentially expressed genes by T-96 compared to control in LCSCs from PRJNA763373. **(B)** GSEA results of KEGG oxidative phosphorylation set from PRJNA763373. **(C)** Heatmap of the differentially expressed mitochondrial coding genes in LCSCs cells with and without T-96 treatment from PRJNA763373. Red stripes represent high-expression genes. Blue stripes represent low-expression genes.

Figure S6

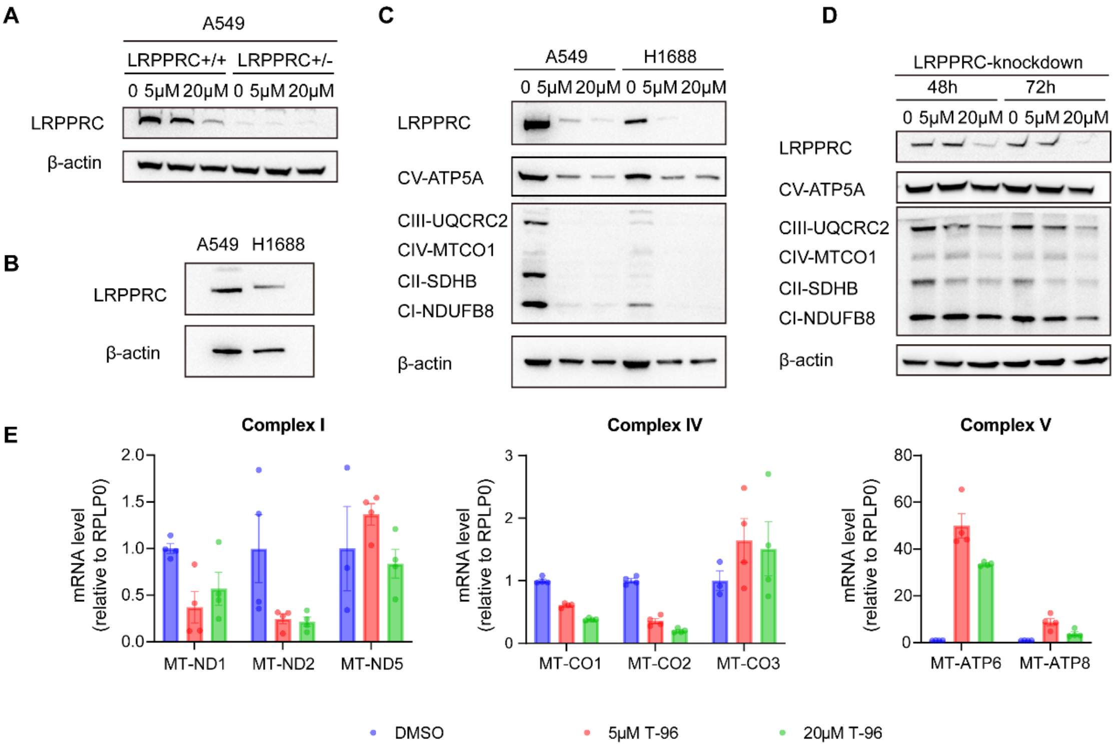

Figure S6. (A) Western blot analysis of LRPPRC in A549-wild type and A549-LRPPRC+/- cells treated with different concentrations of T-96 for 48 hours. **(B)** Western blot analysis of LRPPRC in A549 cells and H1688 cells. **(C)** Western blot analysis of LRPPRC and OXPHOS complex subunits in A549 cells and H1688 cells pretreated with different concentrations of T-96 . **(D)** Western blot analysis of LRPPRC and OXPHOS complex subunits in A549-LRPPRC-knockdown cells pretreated with different concentrations of T-96 . **(D)** mt-mRNA levels of the complex I, III, IV, and V subunits in H1688 cells (mean±s.e.m.; n=4 biological replicates).

Figure S7

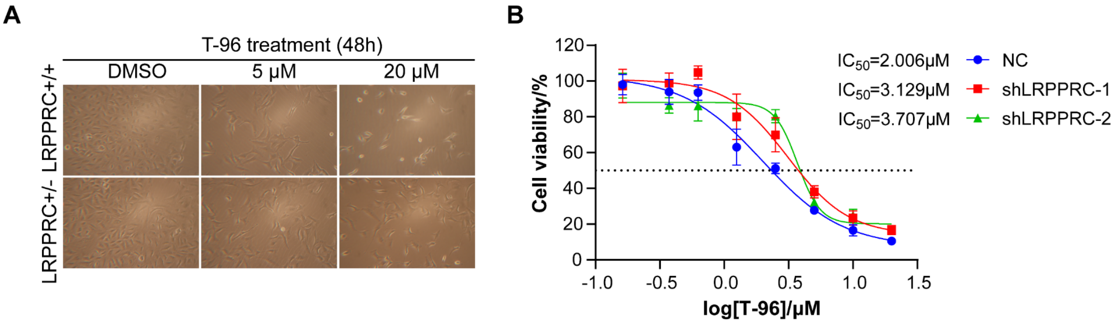

Figure S7. (A) Cell morphology imaging of A549-wild type and A549-LRPPRC+/- cells treated with different concentrations of T-96. **(B)** Effects of T-96 on the viability of A549 NC, shLRPPRC-1, and shLRPPRC-2 cells were evaluated by CCK8 assays. NC, negative control group; shLRPPRC, the short hairpin RNA-mediated knockdown of LRPPRC.

Figure S8

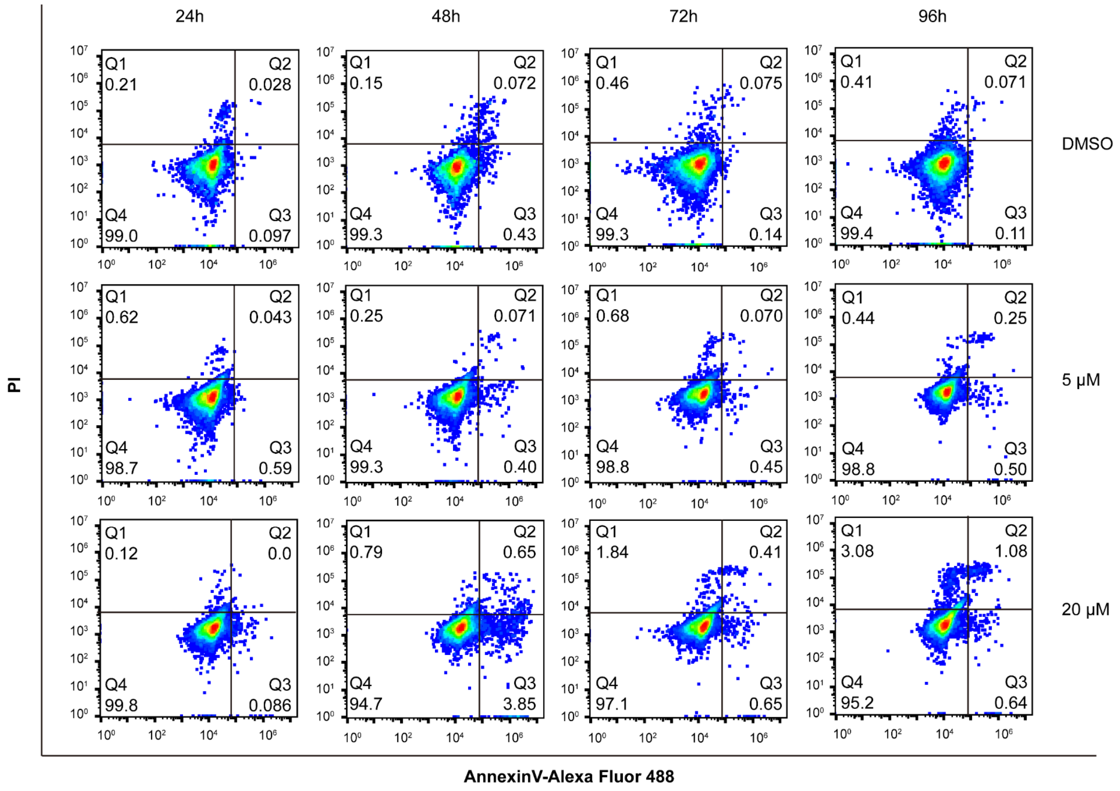

Figure S8. FACS analysis of A549 cell apoptosis. The standard dot plot diagram shows the progression of cell death. Q4, double-negative (Annexin V-Alexa Fluor 488 and PI negative) represented viable cells; Q3, Annexin V-Alexa Fluor 488 positive and PI negative represented apoptotic cells; Q2, Annexin V-Alexa Fluor 488 & PI double-positive represented necrotic cells.

Figure S9

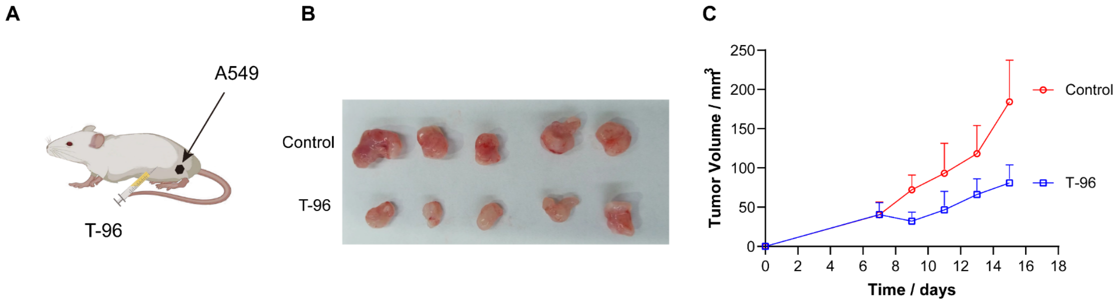

Figure S9. (A) Schematic diagram of T-96 treated mice bearing A549 cells generated subcutaneous tumor. **(B)** Xenograft tumors removed from mice treated with T-96 for 15 days. **(C)** Tumor volume of subcutaneous A549 cells treated with T96 or the control (mean±s.e.m.).

Figure S10

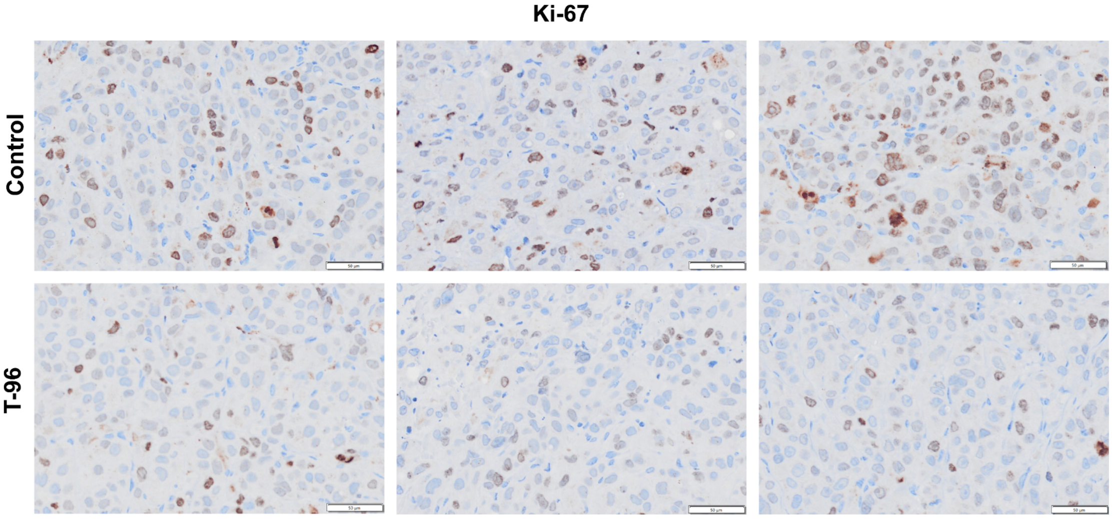

Figure S10. IHC images of Ki-67 in A549 cells xenografts treated with T-96.

Figure S11

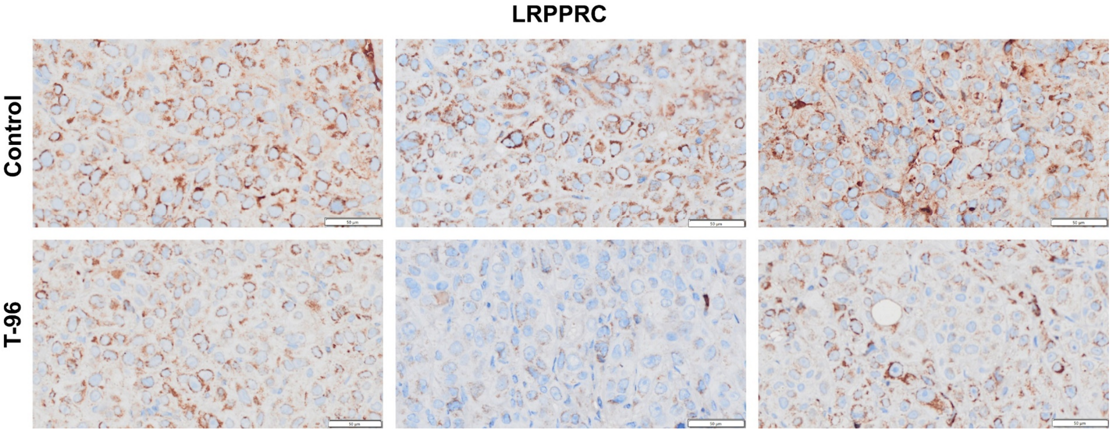

Figure S11. IHC images of LRPPRC in A549 cells xenografts treated with T-96.

Figure S12

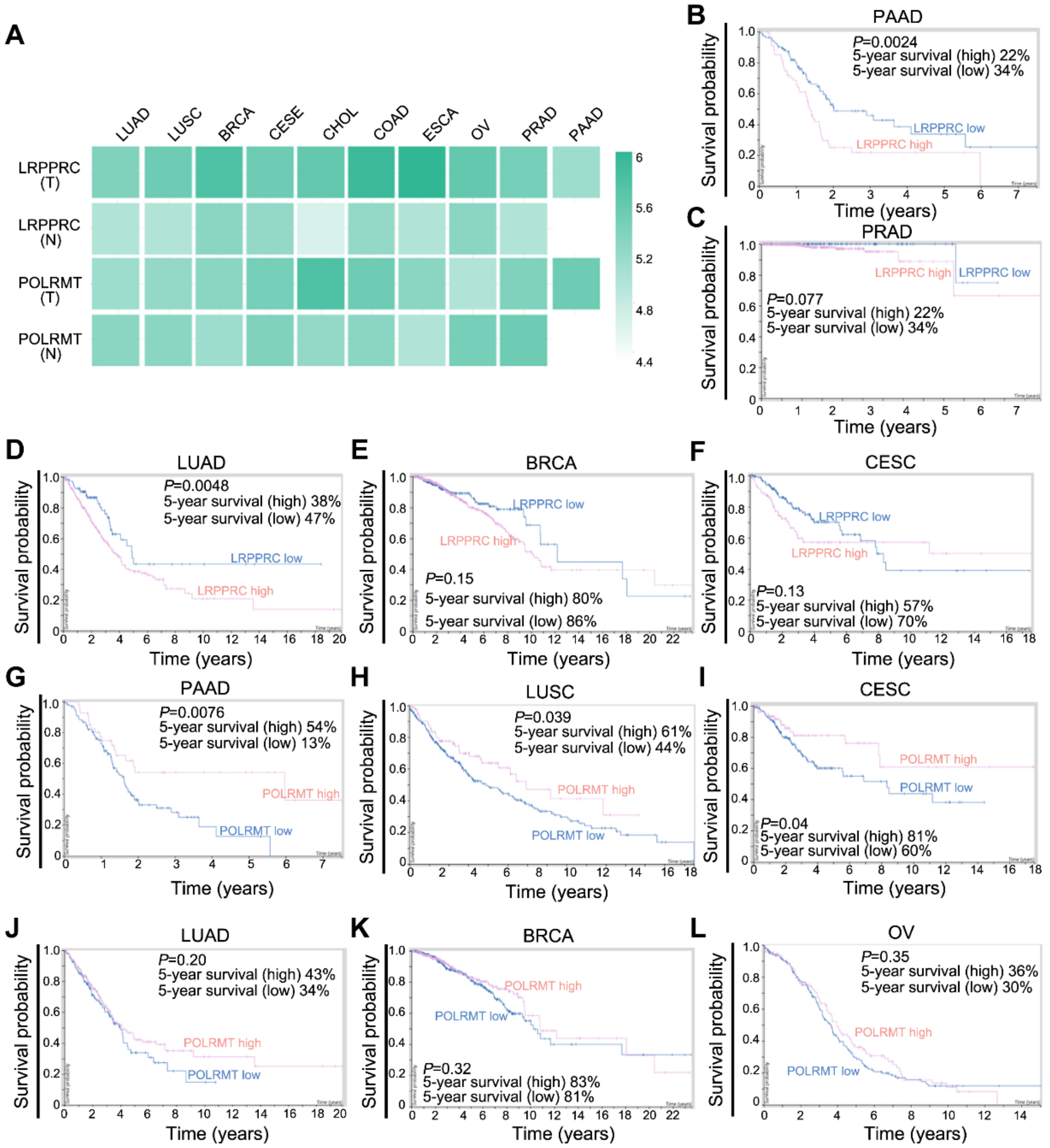

Figure S12. Clinical significance of LRPPRC and POLRMT from TCGA database. (A) Heat map of RNA levels of LRPPRC and POLRMT in the tumor tissues (T) from 10 types of common cancers and corresponding normal tissues (N). The expression of LRPPRC showed upregulated in all these cancers, especially in lung adenocarcinoma and lung squamous cell carcinoma. POLRMT only overexpressed in 3 types of cancers (esophageal cancer, cholangiocarcinoma, and pancreatic cancer) but significantly downregulated in lung adenocarcinoma, lung squamous cell carcinoma, ovarian cancer, and prostate cancer. **(B-L)** Overall survival (OS) analysis of 8 types of cancers with prognostic information. High level of LRPPRC is associated with poor prognosis in 5 types of cancers, including pancreatic cancer (B), prostate cancer (C), lung adenocarcinoma (D), breast cancer (E), and cervical cancer (F). In contrast, patients with a high level of POLRMT showed better prognosis (G-L). The presented OS curves were drawn with the optimal cutoff value of the minimum P value.

Figure S13

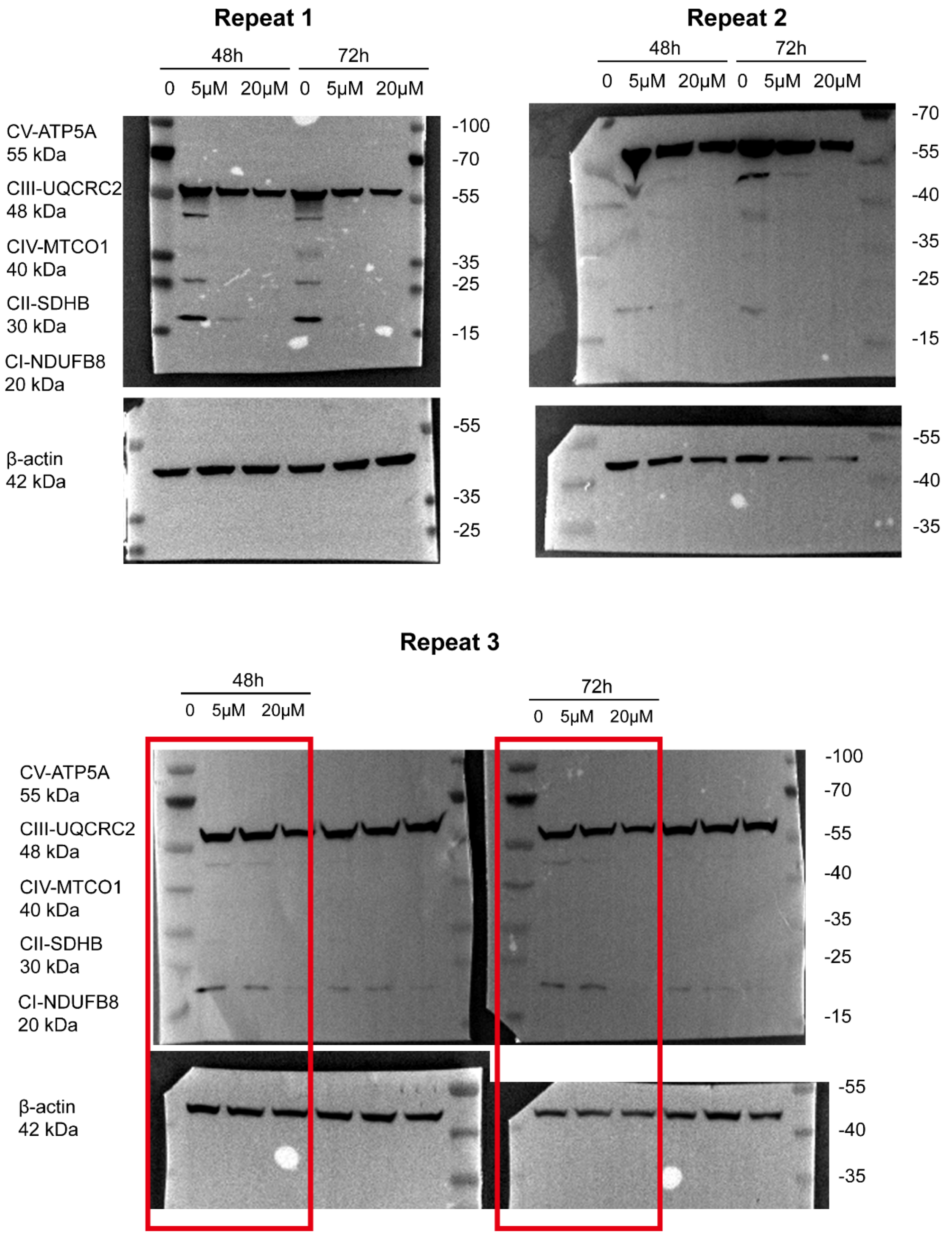

Figure S13. Full original images of Western blotting assays for Figure 2.

Figure S14

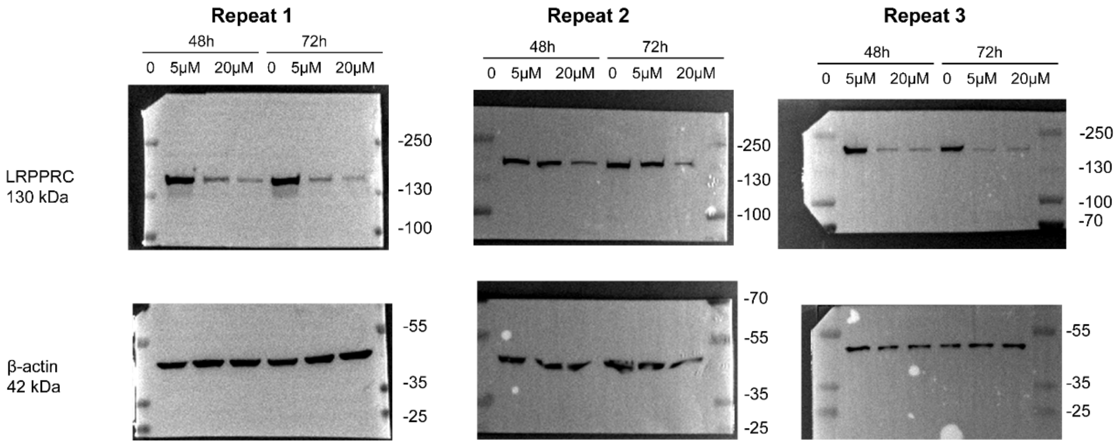

Figure S14. Full original images of Western blotting assays for Figure 3.

SUPPORTING TABLES

Table S1. List of primers for qPCR

Gene	Primer	Sequence
MT-CO1	Forward	CAGTCTACCCTCCCTTAGCA
	Reverse	TGATGGCCCCTAAGATAGAG
MT-CO2	Forward	GACTCCTTGACGTTGACAAT
	Reverse	GGTGAAAGTGGTTTGGTTTA
MT-CO3	Forward	CTCACTATCTGCTTCATC
	Reverse	AAGACCCTCATCAATAGA
MT-ND1	Forward	GAACACCTCTGATTACTC
	Reverse	GTATTCGGCTATGAAGAA
MT-ND2	Forward	TCTCAATCTTATCCATCATAGC
	Reverse	GAATGCGGTAGTAGTTAGG
MT-ND4	Forward	CCTACTCATCGCACTAAT
	Reverse	ATATTAAGTTGTTGGCTCAG
MT-ND5	Forward	TCGCAGGATTTCTCATTACT
	Reverse	GAATCCGAGTATGTTGGAGA
MT-CYB	Forward	CTTACTTCTCTTCCTTCTC
	Reverse	AATTGTGTAGGCGAATAG
MT-ATP6	Forward	ACACTAAAGGACGAACCTGA
	Reverse	GGTGGTTGGTGTAAATGAGT
MT-ATP8	Forward	CCATACTCCTTACACTATTCC
	Reverse	ATGAATGAAGCGAACAGAT
NDUFB8	Forward	CCGCCAAGAAGTATAATATGCGT
	Reverse	TATCCACACGGTTCCTGTTGT
UQCRC2	Forward	TTCAGCAATTTAGGAACCACCC
	Reverse	GGTCACACTTAATTTGCCACCAA
SDHB	Forward	ACAGCTCCCCGTATCAAGAAA
	Reverse	GCATGATCTTCGGAAGGTCAA
ATP5F1A	Forward	AACTGATTATTGGTGACCGACAG
	Reverse	GGCAACAGTGGATCTCTTTTGA
LRPPRC	Forward	AGGTAGTCAGGAGAGTTTGCC
	Reverse	GTGACCCAAGTTGGCACTGA
β-actin	Forward	CATGTACGTTGCTATCCAGGC
	Reverse	CTCCTTAATGTCACGCACGAT
RPLP0	Forward	GTTGCTGGCCAATAAGGTGC
	Reverse	CAGCTGCACATCACTCAGGA

Table S2. List of the top 20 KEGG pathways enriched by T-96 treatment in A549 cells.

ID	Description	p.adjust	qvalue	Count	Fold_Enrichment
hsa04110	Cell cycle	1.44E-19	8.29E-20	147	1.57
hsa04120	Ubiquitin mediated proteolysis	9.38E-18	5.38E-18	132	1.56
hsa04140	Autophagy - animal	9.38E-18	5.38E-18	153	1.52
hsa05014	Amyotrophic lateral sclerosis	4.33E-17	2.48E-17	299	1.36
hsa04141	Protein processing in endoplasmic reticulum	1.41E-16	8.11E-17	152	1.51
hsa04714	Thermogenesis	9.25E-16	5.31E-16	199	1.43
hsa04144	Endocytosis	1.23E-15	7.08E-16	211	1.41
hsa05012	Parkinson disease	2.66E-14	1.53E-14	222	1.38
hsa05016	Huntington disease	3.13E-14	1.80E-14	250	1.35
hsa05010	Alzheimer disease	2.97E-13	1.71E-13	303	1.3
hsa03013	Nucleocytoplasmic transport	1.26E-12	7.23E-13	99	1.54
hsa04932	Non-alcoholic fatty liver disease	1.53E-12	8.76E-13	136	1.46
hsa05208	Chemical carcinogenesis - reactive oxygen species	1.66E-12	9.56E-13	186	1.39
hsa04137	Mitophagy - animal	3.72E-12	2.13E-12	96	1.54
hsa03083	Polycomb repressive complex	1.49E-11	8.58E-12	78	1.58
hsa05131	Shigellosis	1.72E-11	9.90E-12	200	1.35
hsa00190	Oxidative phosphorylation	1.87E-11	1.08E-11	120	1.46
hsa04142	Lysosome	4.01E-11	2.30E-11	115	1.47
hsa05022	Pathways of neurodegeneration - multiple diseases	4.46E-11	2.56E-11	358	1.25
